# Use of Iris Scanning for Biometric Recognition of Healthy Adults Participating in an Ebola Vaccine Trial in the Democratic Republic of the Congo: Mixed Methods Study

**DOI:** 10.2196/28573

**Published:** 2021-08-09

**Authors:** Trésor Zola Matuvanga, Ginger Johnson, Ynke Larivière, Emmanuel Esanga Longomo, Junior Matangila, Vivi Maketa, Bruno Lapika, Patrick Mitashi, Paula Mc Kenna, Jessie De Bie, Jean-Pierre Van Geertruyden, Pierre Van Damme, Hypolite Muhindo Mavoko

**Affiliations:** 1 Department of Tropical Medicine University of Kinshasa Kinshasa the Democratic Republic of the Congo; 2 Global Health Institute University of Antwerp Antwerp Belgium; 3 Centre for the Evaluation of Vaccination University of Antwerp Antwerp Belgium; 4 Department of Public Health Institute of Tropical Medicine Antwerp Belgium; 5 Division Provinciale de la Santé de la Province de la Tshuapa Boende the Democratic Republic of the Congo; 6 Department of Anthropology University of Kinshasa Kinshasa the Democratic Republic of the Congo; 7 Janssen Pharmaceutica NV Beerse Belgium

**Keywords:** biometric identification, iris recognition, vaccine trial, participants' visits, acceptability, feasibility, Democratic Republic of the Congo, mixed methods, Ebola

## Abstract

**Background:**

A partnership between the University of Antwerp and the University of Kinshasa implemented the EBOVAC3 clinical trial with an Ebola vaccine regimen administered to health care provider participants in Tshuapa Province, Democratic Republic of the Congo. This randomized controlled trial was part of an Ebola outbreak preparedness initiative financed through Innovative Medicines Initiative-European Union. The EBOVAC3 clinical trial used iris scan technology to identify all health care provider participants enrolled in the vaccine trial, to ensure that the right participant received the right vaccine at the right visit.

**Objective:**

We aimed to assess the acceptability, accuracy, and feasibility of iris scan technology as an identification method within a population of health care provider participants in a vaccine trial in a remote setting.

**Methods:**

We used a mixed methods study. The acceptability was assessed prior to the trial through 12 focus group discussions (FGDs) and was assessed at enrollment. Feasibility and accuracy research was conducted using a longitudinal trial study design, where iris scanning was compared with the unique study ID card to identify health care provider participants at enrollment and at their follow-up visits.

**Results:**

During the FGDs, health care provider participants were mainly concerned about the iris scan technology causing physical problems to their eyes or exposing them to spiritual problems through sorcery. However, 99% (85/86; 95% CI 97.1-100.0) of health care provider participants in the FGDs agreed to be identified by the iris scan. Also, at enrollment, 99.0% (692/699; 95% CI 98.2-99.7) of health care provider participants accepted to be identified by iris scan. Iris scan technology correctly identified 93.1% (636/683; 95% CI 91.2-95.0) of the participants returning for scheduled follow-up visits. The iris scanning operation lasted 2 minutes or less for 96.0% (656/683; 95% CI 94.6-97.5), and 1 attempt was enough to identify the majority of study participants (475/683, 69.5%; 95% CI 66.1-73.0).

**Conclusions:**

Iris scans are highly acceptable as an identification tool in a clinical trial for health care provider participants in a remote setting. Its operationalization during the trial demonstrated a high level of accuracy that can reliably identify individuals. Iris scanning is found to be feasible in clinical trials but requires a trained operator to reduce the duration and the number of attempts to identify a participant.

**Trial Registration:**

ClinicalTrials.gov NCT04186000; https://clinicaltrials.gov/ct2/show/NCT04186000

## Introduction

Identification and recognition of study participants in a clinical trial—during the process of recruitment and during follow-up visits—is a growing issue [1]. Conventional methods for the recognition of participants in health facilities may include patient name, date of birth, government identity card with photo, and phone number [2-5]. However, these methods are not always reliable or accurate [5]. For example, identity cards can be stolen or forgotten, and there is a risk of assigning a participant's ID (intentionally or unintentionally) to another participant during a study visit. Some participants may share their ID card number with a family member with a similar physical resemblance, if they are unable or unwilling to keep to their appointment time. In clinical trials, efficacy and safety data such as (serious) adverse events, are repeatedly assessed through anamneses, physical examinations, and biological samples during different visits, possibly, over a long period of time. Thus, participant enrollment and identification are essential steps to ensure that all data collected are unique and that neither the participant nor the visit has been misidentified [1,2]. A biometric identification method coupled with a unique participant ID number could mitigate the occurrence of mistakes made using conventional methods during initial and follow-up clinical trial visits [3].

Biometric technology confirms the physical presence of the person by assessing unique physical or behavioral characteristics that cannot be borrowed, stolen, or forgotten. Such technology uses matching algorithms or artificial intelligence for identifying the particular feature [3,6,7]. A number of biometric identifiers, including physical traits (eg, fingerprint; face; palm; cornea; iris; thermogram of the body, face or ear; and DNA) or behavioral traits (eg, signature, voice, typing dynamic, smell, and walk pattern) have demonstrated technical feasibility in various studies [6-10]. Biometric identification systems have many advantages over more conventional methods of identification, such as easier fraud detection and more accuracy for face recognition than photographs. Therefore, biometric identification is increasingly used worldwide in various fields to recognize individuals and secure their data (eg, during elections, at airports, or for criminal detection) [6].

Irises are an ideal part of the body for biometric identification. The iris is flat and has a fine texture and geometric configuration determined randomly upon embryogenesis [3,10,11]. It is a unique, permanent, and universal “biometric signature” present throughout a person’s lifespan, which is covered by a highly transparent and sensitive membrane that makes it distinctive from other biometric methods [1,2]. A human iris is always stable, irrespective of age [10]. This is in contrast to the fingerprint structure—the most widespread biometric method of identification—that varies during childhood and only becomes stable after many years [5,9]. Fingerprinting also carries additional risks such as spreading some infectious diseases, since it requires the participant (and, sometimes, the operator) to come in physical contact with the fingerprint device. Identical (ie, monozygotic) twins were found to have higher similarities of fingerprint patterns compared with nonidentical twins [4,5]. Iris scanning is feasible under most circumstances, as it can be carried out from anywhere between 10 cm to several meters away from the eye, and results are generally available within 30 seconds [7,12]. Even genetically similar people have entirely independent irises; thus, iris scanning recognition avoids misidentification of identical twins [3-5]. However, iris recognition may be challenging for people who suffer from diabetes or any other iris disease [4,5]. Moreover, the accuracy of the scanning devices can be affected by unusual light effects, in comparison with fingerprinting [2,5].

Iris scans may offer one of the most secure strategies of authentication and recognition in clinical trials [7]. Iris-based biometric systems have demonstrated a promising performance during the process of recognition, with an average time (during initial clinical trial enrollment) of less than 2 minutes and a sensitivity rate of at least 86% [8,11,12]. In Kenya, an iris scan sensitivity or accuracy rate of 95% was found in HIV and tuberculosis patients during routine hospital consultations [8]. This was better than fingerprint biometric recognition found in Ghana (68.7%) or in Uganda (75.5%) [11,12]. Thus, use of iris scan technology can substantially reduce the possibility for fraud and abuse within a clinical trial [3,9,10]. Lastly, it has a high acceptance rate, with very low false-match and rejection rates [1,2].

Despite its attractive design features, there is little information available about the acceptability of iris scan technology for the general public, especially, information on how it varies across and within countries. Acceptability within a population may depend on many factors such as positive perception, confidence, and constraints presented against the use of iris scans. For example, in one of the few studies available, a survey was conducted in Australia on the willingness of the general population to use biometric security technologies; it found that 61% of the population would accept fingerprints, whereas only 41% would accept iris scan recognition [6]. In California, 72% of participants preferred an identification by fingerprint [9]. A remarkable acceptability rate of iris scanning itself (98.9%) was noted in a survey on an identification system of routine clinic services in Kenya [8].

As part of an ongoing Ebola vaccine clinical trial (EBOVAC3, study protocol number VAC52150EBL2007, clinicaltrials.gov identifier NCT04186000) [13], we assessed acceptability, accuracy, and feasibility of iris scan technology as a biometric identification method within a population of health care provider participants in a remote setting.

## Methods

### Study Design

A mixed methods study design assessed the acceptability, accuracy, and feasibility of the iris scan as a biometric identification tool in the Ebola vaccine trial in Boende, Tshuapa province, Democratic Republic of the Congo (DRC). Acceptability was assessed through focus group discussions (FGDs) with volunteering health care provider participants and via a survey with a structured questionnaire. Feasibility and accuracy research was conducted using a longitudinal study design, where iris scanning was used to uniquely identify health care provider participants at enrollment and at their follow-up visits in the clinical trial. Accuracy and feasibility studies were conducted from December 2019 to April 2020, from the second participant visit (day 57) until the third participant visit to the study site (day 78) (Multimedia Appendix 1).

### Participants and Recruitment Procedures

For the qualitative acceptability assessment, study participants were selected using purposive, nonprobability sampling. A total of 86 participants were enrolled in 12 focus FGDs (Multimedia Appendix 1). For the FGDs, we selected key informants from the following stakeholder groups: nurses, community health workers, laboratory technicians, medical doctors, first-aid officers, birth attendants, and hospital cleaners. All recruited health care provider participants worked at the reference hospital or health centers within the Boende District, with the exception of nurses, who worked in health centers throughout Tshuapa Province. Research activities occurred at 5 sites, all located in the Boende Health Zone: Boende General Hospital (ie, Hôpital Général de Référence de Boende), Boende Catholic Mission, N’sele Health Center (ie, Centre de Santé Boende II N’sele), Motema Mosantu Health Center (ie, Centre de Santé Motema Mosantu), and Communauté des Disciples du Christ au Congo Health Center (ie, Centre de Santé CDCC).

For the quantitative study component (assessing acceptability, accuracy, and feasibility), all health care provider participants enrolled in the clinical study (N=699) were included. All participants were health care provider participants working in the Boende Health District. Their workstations were located between 0 kilometers and 50 kilometers away from Boende General Hospital.

### Ethical Approval

Research was conducted in line with the prevailing ethical principles of socio-behavioral studies with human populations to protect the rights and welfare of all participants. Permission to undertake the acceptability (qualitative) study was granted by the DRC National Ethics Committee for Health (reference 93/CNES/BN/PMMF/2019), the Institute of Tropical Medicine, Belgium (reference 1293/19), and the University of Antwerp, Belgium (reference 19/14/188). Permission for the accuracy and feasibility (quantitative) study, collected during the course of the ongoing clinical trial, was granted by the DRC Ethical National Committee (reference 137/CNES/BN/PMMF/2019).

### Data Collection and Informed Consent

#### Pretrial Study

Based on a literature review, a topic guide was developed highlighting potential key issues with regard to the acceptance of new technologies among health care providers in DRC. This review formed the basis for the design of the FGD tool, which included questions and probes focusing on the background of health care provider participants and their role in the community, their acceptance of new technologies and communication strategies, and their recommendations for appropriate identification and communication tools with trial participants (Multimedia Appendix 1). University of Antwerp and University of Kinshasa (UNIKIN) team members reviewed and refined the research tools prior to their finalization and implementation. Specific questions and probes were reviewed and refined during the research period, in light of arising themes (eg, an ongoing Monkeypox vaccine trial in Tshuapa at the time of data collection) [14].

Key topics were addressed in each discussion, to allow for generalization of themes across participant groups. The research was deliberately designed to facilitate input from multiple health care provider participant stakeholders in a step-wise manner, so that issues raised by one group of participants were also discussed with other participant groups to assist with triangulation of data. At the start of each discussion, it was made clear to all potential participants that their involvement was optional and voluntary. The study’s consent form was presented and explained in detail, and all participants’ questions were answered prior to beginning data collection. Informed consent was given verbally. All FGDs were conducted in either French or Lingala, depending on the linguistic preferences of participants. FGDs lasted for approximately 60-80 minutes. Audio recordings were made, along with field notes, which served as the basis for a thematic analysis of data. Concurrent to FGDs, acceptability was (quantitatively) defined as the number of participants agreeing to iris scanning as a proportion of all the individuals approached. Reasons for declining iris scanning were elicited from participants.

#### Intratrial Study

Accuracy was measured by the rate of successful recognition of study participants (percentage of participants recognized by the iris scan) during the participants’ third visit (day 78). This was achieved by cross-referencing the output of the iris scan with the clinical trial identity card of each participant, to make sure that it was indeed the correct study participants returning on their corresponding scheduled visit dates. We considered it a wrong match when registered participants returned for their next visit and the system gave details of more than one possible identification record. Feasibility was measured by how long (ie, duration of operation based on ranges ≤1 min, 1 min 1 s-1 min 30 s, 1 min 31 s-2 min, 2 min 1 s-2 min 30 s, or ≥2 min 30 s; Table 1, Table 2, and Table 3) the iris scanning device took to recognize each study participant in the EBL2007 Ebola vaccine trial during the second and the third visit (ie, day 57 and day 78, respectively) and the number of scanning attempts that were required by the iris scan operator or by the iris scan devices (ie, tablet, scanner, server, and Wi-Fi connection between server and tablet) during these same visits. The duration of operation included the time for the biometric tablet to capture the iris image, identity photo, and demographics and the time it took to link these data with the local server. An assessment of time to recognize each study participant at their third visit was recorded by the operator. It is important to note that a problem was encountered during the first study visit (day 1), where all vaccinated participants who received their first vaccine dose on that day should have had their demographic information and iris scan recognition registered on the server. However, these data were lost due to a manual error that occurred when attempting to save all of the data collected for this visit, resulting in the loss of participant demographic and biometric data. This error was corrected during the second visit (day 57), when all participant data were re-entered (Multimedia Appendix 1).

**Table 1:** Duration of the initial iris scan process in the EBL2007 clinical trial in Boende, Tshuapa Province, Democratic Republic of the Congo (N=683).

Duration of iris scanning operation to record subjects	Frequency, n (%)	95% CI	Cumulative percentage, %
0 s-1 min	280 (41.0)	37.3-44.7	41.0
1 min 1 s-1 min 30 s	332 (48.6)	44.9-52.4	89.6
1 min 31 s-2 min	44 (6.5)	4.6-8.3	96.1
2 min 1 s-2 min 30 s	24 (3.5)	2.1-4.9	99.6
≥2 min 30 s	3 (0.4)	0.0-0.9	100

**Table 2:** Iris scan attempts at the second visit in the EBL2007 clinical trial (N=683).

Number of iris scanning attempts	Frequency, n (%)	95% CI	Cumulative percentage, %
Once	475 (69.6)	66.1-73.0	69.5
Twice	149 (21.8)	18.7-24.9	91.4
Three times	59 (8.6)	6.5-10.8	100

**Table 3:** Duration of the iris scan process during the third visit in the EBL2007 clinical trial (N=683).

Duration of the operation at the third visit	Frequency, n (%)	95% CI	Cumulative percent, %
0 s-1 min	665 (97.4)	96.1-98.6	97.4
1 min 1 s-1 min 30 s	1 (0.1)	0.0-0.4	97.5
1 min 31 s-2 min	9 (1.3)	0.5-2.1	98.8
2 min 1 s-2 min 30 s	5 (0.7)	0.1-1.3	99.5
≥2 min 30 s	3 (0.5)	0.0-1.0	100

### Equipment and Procedures

The iris scan operator, a trained and authorized study staff member, used an iris camera (Iritech, Irishield Monocular Fairfax, VA 22030, United States), and a tablet (Samsung Tab Active 2, Suwon, South Korea) connected via Wi-Fi to a local ruggedized server (Cincoze DX-1100, New Taipei City, Taiwan) located approximately 10 meters from his physical location. An external hard drive for backing up the iris scanning database was located nearby as well. A biometric user interface running on the Samsung Tab Active 2 was designed by Janssen Pharmaceutica NV Beerse, Belgium. In addition to the iris scan, the operator captured demographic data on the biometric tablet, such as gender, year of birth, participant ID, passport photo, contact telephone number, date, and time stamps of the iris scan. Activities performed on the tablet were captured in an audit trail with date and time stamps. The biometric tablet allowed the operator to assess whether administration of the second vaccine dose (administered on day 57) as well as the blood collection during the third visit (day 78) were in the predefined visit windows or not. To capture the irises, the operator stood in front of the study participant and held a camera in their right hand and a tablet in their left hand. Participants were seated so that their head and body were vertically aligned. The distance between the iris and the camera ranged from 3-10 cm.

### Data Analysis

#### Pretrial Study

At the conclusion of the research activities, the lead qualitative researcher, GJ, a medical anthropologist, transcribed notes, alongside rereviewing audio files to compile data for review and verification. Notes were typed in either English or French. A preliminary analysis of qualitative data was conducted throughout the data-collection process. The lead researcher, GJ, was responsible for all thematic analysis of qualitative data. Dominant themes were identified through the systematic review of FGD audio and transcribed notes. The occurrence and reoccurrence of salient concepts were labelled throughout, and emerging trends were critically analyzed according to the research objectives and topic guide. An appointed research member, TZ, was additionally responsible for maintaining the quantitative survey database with the acceptance rate of iris scanning at the end of discussions.

#### Intratrial Study

Data with regard to the accuracy and feasibility were collected in an Excel spreadsheet. The dataset was checked for any inconsistencies such as duplicates and then processed using Excel to synthesize the results in terms of proportions.

## Results

### Pretrial Study: Acceptability and Concerns About the Iris Scan

Data collection and in-country fieldwork were conducted in April 2019. Overall, acceptance (85/86, 99%; 95% CI 97.1-100.0) of the iris scan technology was widespread. As stated by one nurse:

For me, I accept [iris scanning], because I know it is a process that is being used to cast away Ebola, and we want this disease to leave.

However, another research participant, also a nurse, refused to have her picture taken while consenting to have her iris scanned, on the justification that the picture may cause problems with her church superiors.

FGD participants voiced some primary concerns about the iris scan. The concern that the iris scan may cause physical problems to their eyes was widespread, across all stakeholder groups and education levels. As stated by one community health worker:

We are agreeing with what you say, but we are afraid with the use of the eye scan, because we fear it may cause problems with our eyes.

Similarly, one birth attendant stated:

We are asking because the eyes are the life of the people, so, after using the eye scan, will there be some problems, for us, with our eyes?

Participants often associated the extended duration of some scans as harmful to their eyes due to the light emitted by the scanner. The research team often heard participants asking:

Will the scanner disturb the eyes with the [light] rays, in relation to the duration?

Participants also asked:

Are you sure that this scan will not hurt our eyes?

The pretrial acceptability study, therefore, noted that there was a higher risk for the participants enrolled in the trial to link any vision loss to the iris scan. In fact, even if a participant consented to an eye scan at the time of the vaccination as indicated by the quantitative survey (ie “we are agreeing with you*”*), any problems pertaining to eyes (through naturally occurring means) could later be associated with the iris scan. This is illustrated by the following exchange with a laboratory technician:

We are using the microscope, and we are suffering from our eyes because of looking through the microscope, so maybe we will have a problem in the long-term with our eyes...our first thought will be that the technology caused this problem, so this is why we need a very good explanation, so that we know it is not the technology that is causing the problem.

A second concern was wondering, will the “iris scan...expose me to spiritual problems through sorcery?” This was discussed by most stakeholder groups as primarily a problem for “those who are not learned” or those who belong to churches that reject vaccination (ie, “some churches here that are proving to the population that they should not receive a vaccination*”*). For example, discussions with doctors, nurses, and laboratory technicians regarding persons who may be concerned about the potential of the technology to open them up to witchcraft, often started with the phrase:

For us, there is no problem [with the iris scan], but other people will need to be sensitized to accept…[T]he education level of the population is very low. If you use the eye scan, they may think you are trying to make trouble through their eyes.

By using the phrase “us,” health care provider participants are referencing persons such as themselves who are well-educated health professionals. This sentiment did not often extend to “other” stakeholder groups (eg, community health workers) with a lower-level of education. This comment from a laboratory technician is illustrative:

By using the eye scan, many people will be having a bad [thought] that the eye scan will cause trouble with the eyes, and people will run away,...because, if you are using the eye scanner, they will think you are putting something into their eyes.

The pretrial acceptability study, therefore, noted that regardless of whether or not the health care provider participants who are enrolled in the trial harbor suspicions about the technology with regard to witchcraft, they are embedded in the larger cultural and religious communities of Tshuapa, who are likely to have such concerns. As such, trial organizers should be aware of, and have a communication plan prepared for, the potential myths and rumors that may manifest in Tshuapa, which associate the iris scan with the evil intentions of witchcraft.

Three types of identification were familiar and considered to adequately identify vaccine recipients: ID cards (containing name, address, phone number, etc), thumbprints or fingerprints, and facial photographs. An ID card as a method of identification was used in the Monkeypox vaccine trial, which was still occurring in the area while the qualitative pre-EBOVAC3 trial activities were ongoing. This was used to identify the trial enrollees. Several doctors familiar with the Monkeypox vaccine trial felt it may be confusing for some EBOVAC3 participants to be requested to have their eye scanned as a method of identification, given their familiarity with a different method as established by the recent Monkeypox vaccine trial. Participants also felt strongly that the use of a facial photograph by itself (without scanning both eyes) was a sufficient method to identify individual persons. As stated by one nurse:

If the iris scan is just a picture of the eye, why not just take a picture of the person? This is also a positive way to identify them, which does not take so much time.

A community health worker similarly stated:

I can change my clothes, but I can’t change my face.

In general, participants were confused as to why 3 pictures—1 of their face and 1 of each eye—were necessary as a method of identification. While the iris scan technology was not rejected by most participants, many favored the use of ID cards plus facial photographs as a positive method of identification. Preference for photos of their face rather than a scan of each eye was due to considering photos as less invasive and less time-consuming but equally positive as a way to identify an individual.

### Intratrial Study: Acceptance, Accuracy, and Feasibility of Iris Scan Identification Technology

It was noted that, of 699 participants enrolled, 99.0% (692/699; 95% CI 98.2-99.7) had given consent to be identified by the iris scan technology. Thus, 7/699 (1.0%; 95% CI 0.3-1.7) of the participants refused. Various reasons were given for refusing, but most of them argued about the fear of alterations to their visual acuity over time (Table 4). Among the participants who agreed to have their iris scanned, 0.9% (6/692; 95% CI 0.2-1.6) did not return for the second or third visits. In addition, iris scan data of 0.4% (3/692; 95% CI 0.0-0.9) of the participants were not properly entered in the database at the second visit due to inattention by the iris scan operator during the registration process of inputting data in the server. As a result, the quantitative survey conducted for the accuracy and feasibility study was only possible for 683/692 (98.7%) of participants who agreed to be identified by an iris scan during their initial clinical trial visits.

Capturing a successful and quick iris scan is a process requiring both a participant who is willing to follow operator instructions (eg, face forward, chin down, etc) and a skilled operator capable of balancing the tablet in one hand while successfully locating the iris with the scanning device in the other hand. It often took more than one attempt to receive feedback on the tablet screen that a participant’s irises were correctly scanned in the iris scan server (Table 2).

During the process of rerecording each participant’s iris scans for both the first and second visit of the clinical trial, the duration of the operation ranged from 1 minute 1 second to 1 minute 30 seconds, for the majority of study participants (332/683, 48.6%; Table 1). Capturing a successful image of the iris often took several seconds and required multiple manipulations of participants’ face and body by the iris scan operator, in order to obtain a successful reading (Table 1). This concern seemed to exacerbate participant conclusions that the eye scan was taking too long and potentially causing long-term damage to their eyes. The process of recording study participants by scanning their irises, capturing a photo, and entering their demographic data into the tablet lasted 2 minutes or less for 96.0% (656/683; 95% CI 94.6-97.5) of participants. At the third visit, it took less than 1 minute for 97.4% (665/683; 95% CI 96.1-98.6) of participants to be authenticated. Overall accuracy of the iris scan, calculated by the percentage of successful iris scanning recognitions on the third visit, was 93.1% (636/683; 95% CI 91.2-95.0).

**Table 4:** Participants in the EBL2007 clinical trial who refused to be identified by an iris scan.

Gender	Iris scan performed? (yes or no)	Reason for denial of iris scan
Male	No	Fears the scanner will cause defective vision in the future
Male	No	Deteriorated vision prior to enrollment in the clinical trial and fears the scanner will further damage his eyes
Female	No	Fear of the iris scanning tools and devices
Male	No	Fears the scanner will cause defective vision in the future
Female	No	Fear of the iris scanning tools and devices; fears the scanner will cause defective vision in the future
Male	No	Fear of the iris scanning tools and devices
Male	No	Fears the scanner will cause defective vision in the future

## Discussion

### Principal Findings

In general, iris scanning as a biometric technology for identifying participants in a clinical trial was acceptable, feasible, and accurate. A high acceptability rate (99.1% pretrial; 99.0% intratrial) of biometric identification via iris scanning was noted among the health care provider participants. This remarkable rate of acceptance was similar to the one found in the quantitative survey conducted prior to the implementation of this technology for the clinical trial.

Results from the quantitative survey should be interpreted with care, as health care provider participants may not be representative of the general population of Tshuapa province or elsewhere. The qualitative data presented here describe a more nuanced picture of technology acceptance (eg, concerns over physical or spiritual problems from the iris scan) than the reported quantitative survey results alone. Prior to starting the clinical trial, the quantitative survey conducted among potential trial participants found that less than 1.0% of them refused the irises scans and preferred other identification methods such as simply capturing a photo on a participant's card or registering fingerprints. This low refusal rate was confirmed during the implementation of the trial. This is an illustration of the need to anticipate risk perceptions in a community of potential clinical trial participants via a prior acceptability study, in order to determine beforehand whether or not that community is ready to use an innovative biometric identification technology. To our knowledge, this is the first study in sub‑Saharan Africa demonstrating the use of iris recognition in a clinical trial involving an adult population. Our high acceptability (99.1% pretrial; 99.0% intratrial) is comparable with other observational studies using iris scans in Kenya and Brazil [8,15]. This is likely because potential participants were already briefed on the value of using iris scans in the trial prior to the start of the study through a previous workshop. The FGDs conducted during the pretrial qualitative study helped clinical trial investigators “empty all pockets of fear” with regard to the use of this innovative technology. Furthermore, demonstrations of the functionality of this tool, the explanations given during the qualitative survey, and the ability to explore participants’ potential fears and concerns about the technology through FGDs likely had an influence on the willingness of health care provider participants to accept the iris scan as an identification technique.

Various reasons were given by trial participants for potential refusals of the iris scan, but most were fearful that their visual acuity would be altered over time. Fears associated with a new and unknown technology needed to be overcome, not only by volunteers but also by the iris scan operator, who struggled at the start of the trial with using a new technology and making sure that all participant details were recorded accurately and quickly (to limit participant fears). That is, while implementing the iris scan in the clinical trial, several issues did arise with regard to some extended wait times for receiving the feedback that a good-quality iris scan stamp was properly recorded, before the ability to enter participants’ other demographic details. In addition, the use of the tablet to instantaneously capture an image of the participant’s face, prior to proceeding to scan the eye, caused participants to conclude that the eye scan was taking too long. Capturing an image of the participant’s face was always quickly and immediately successful, without any special posturing by the participant. However, capturing a successful image of the iris often took several seconds and required multiple manipulations of their face and body by the iris scan operator in order to obtain a successful reading. This sometimes caused whispering and fatigue in the queue of health care provider participants, who were often impatient about waiting in line for their turn, especially, when the iris scanner took 2 minutes or more to successfully capture 1 iris scan. The longer duration at the second visit was, however, due to data re-entering that had to be performed for both visit 1 and visit 2. That would likely not have been the case if the manual error had not occurred after the day-1 visit, which could have saved time and speculations from the participants. During the third visit, things were easier for the iris scan operator, as only one scan of an iris was enough for the system to provide the picture and the appointment window of the participant. Continuous practice by the operator is, therefore, important for the success of using this technology.

Finally, quantitative research demonstrated that iris scanning technology can be used effectively in clinical trials in resource-poor countries. An accuracy rate of 93.1% in this study is better, compared with the 85% accuracy reported in Brazil [15]. However, the accuracy rate in Kenya was even higher, at 95% [8]. With these appreciable accuracy rates, iris scan technology demonstrates the importance of scaling it up in the future, for widespread use in clinical trials and for the automation of subjects’ identification processes. The time duration required to capture the iris scan and other related information of health care provider participants at enrollment is similar to that reported in Brazil (less than 2 minutes) [15]. This time is shorter than the average of 4 minutes reported in Kenya [8]. It is understood that this time depends on the amount of information needed for each person included in the study, and that this is a factor that influences the recording time at recruitment. It should also be pointed out that the accuracy rate of 93.1% may have been underestimated, given that a failed recognition was scored even when the correct matching profile was presented along with other possible matching profiles upon completion of the iris-recognition process. The training of scanning operators, for the steps to take if during the matching process multiple profiles are offered, is more important here and, to a lesser degree, an issue of practice.

Some weaknesses were found that could be attributed either to the operator or to the iris scan system. With regard to the operators, if they did not scan both irises with equal precision (after each iris scan, the biometric tool showed the iris scan precision with color codes green [high], orange [medium], or red [low precision]), the biometrical tool sometimes provided several possible participant matches as an output. Based on a photograph entered at the beginning of the trial, the operator could then select the correct participant. Similarly, an issue sometimes occurred when the operators did not correctly enter the study ID for the participant in front of them, which is the basis for the pop-ups of the participant information on the tablet. A loss of information is also possible, as was the case after the first visit of this study, if the operator or the site does not pay attention to the standard operating procedure of the system (eg, how to save the recorded information). This would constitute a deadlock to identify participants for the future visits. In the event of a possible false match or a correct match, the identity of each participant was to be double-checked using the profile picture and biographical data also entered into the biometric tool, as well as with a participant ID card. Yet, cross-referencing outputs of a biometric tool to an ID card may present a risk in underestimating accuracy of the biometric tool. In fact, the use of the ID card as a reference, which can be tampered with, can compromise the benefit of the biometric tool in detecting fraud [4].

Nonetheless, it is worth mentioning that during subsequent visits, the iris scan allowed detection of some cases of fraud attempts. For example, some people not enrolled in the trial tried to come on a scheduled visit to replace a relative. In addition, a few study participants attempted to falsify their ID numbers in order to change the study’s activity schedule (ie, vaccination or blood sample collection). In these cases, the iris scanning system was able to catch the attempted fraud. Moreover, the Ebola vaccine trial has quite a long follow-up period. This highlights the relevance of using this technology to correctly identify the clinical trial participants, to make sure, for example, that a blood sample is collected from participants who actually received the study intervention and not from their relatives.

It seems important to consider a qualitative study that measures trial participants’ perceptions after identification by an iris scan, as these perceptions can add value. This would provide a better understanding of the contours of the level of acceptability among health care provider participants in a vaccine trial.

During the assessment of acceptability, accuracy, and feasibility of the iris scan system, nearly no technical issues were encountered. The equipment that was used had the advantage of lacking dependency on internet connection, as the device was connected to the server via a local Wi-Fi connection. In a few occasions where a technical problem occurred, it was troubleshooted automatically by rebooting or bringing the tablet closer to the server. Both the tablet and the server needed a power supply. Hence, its implementation in a remote area should take this into account beforehand. In the Ebola vaccine trial in Boende, a generator was running permanently onsite, and uninterrupted power supplies were available as back-ups.

### Conclusions

Identification through iris scanning is an innovative technology that was found to be acceptable, accurate, and feasible with health care provider participants in a remote setting. This biotechnical tool takes little additional time, can automate the process of identifying subjects in a clinical study, and can quickly recall relevant information in relation to trial appointments. Thus, it helps to guarantee the quality of data. Sensitization of investigators and potential study participants and their communities is a necessary prerequisite to successfully introduce this promising technology in trials conducted in low- and middle-incomes countries. We hope that this paper will, therefore, spark the idea of proposing further explorations in the field of biometric identification technology. Then, solutions could be found for the difficulties encountered here, to further leverage performance of the iris scan as a fast and reliable biometric method to implement in clinical trials.

## Appendix

Multimedia Appendix 1 Completed and planned events of EBL2007 clinical trial, focus group discussion stakeholder groups, and research tool.

## Abbreviations

DRC: Democratic Republic of the Congo
FGD: focus group discussion
