# Supplementary material for: Use of Iris Scanning for Biometric Recognition of Healthy Adults Participating in an Ebola Vaccine Trial in the Democratic Republic of the Congo: Mixed Methods Study
Source: J Med Internet Res. 2021 Aug 9;23(8):e28573. doi: 10.2196/28573 (PMC8386356; doi:10.2196/28573)
Supplement: Multimedia Appendix 1 [file jmir_v23i8e28573_app1.pptx]

## Slide 1
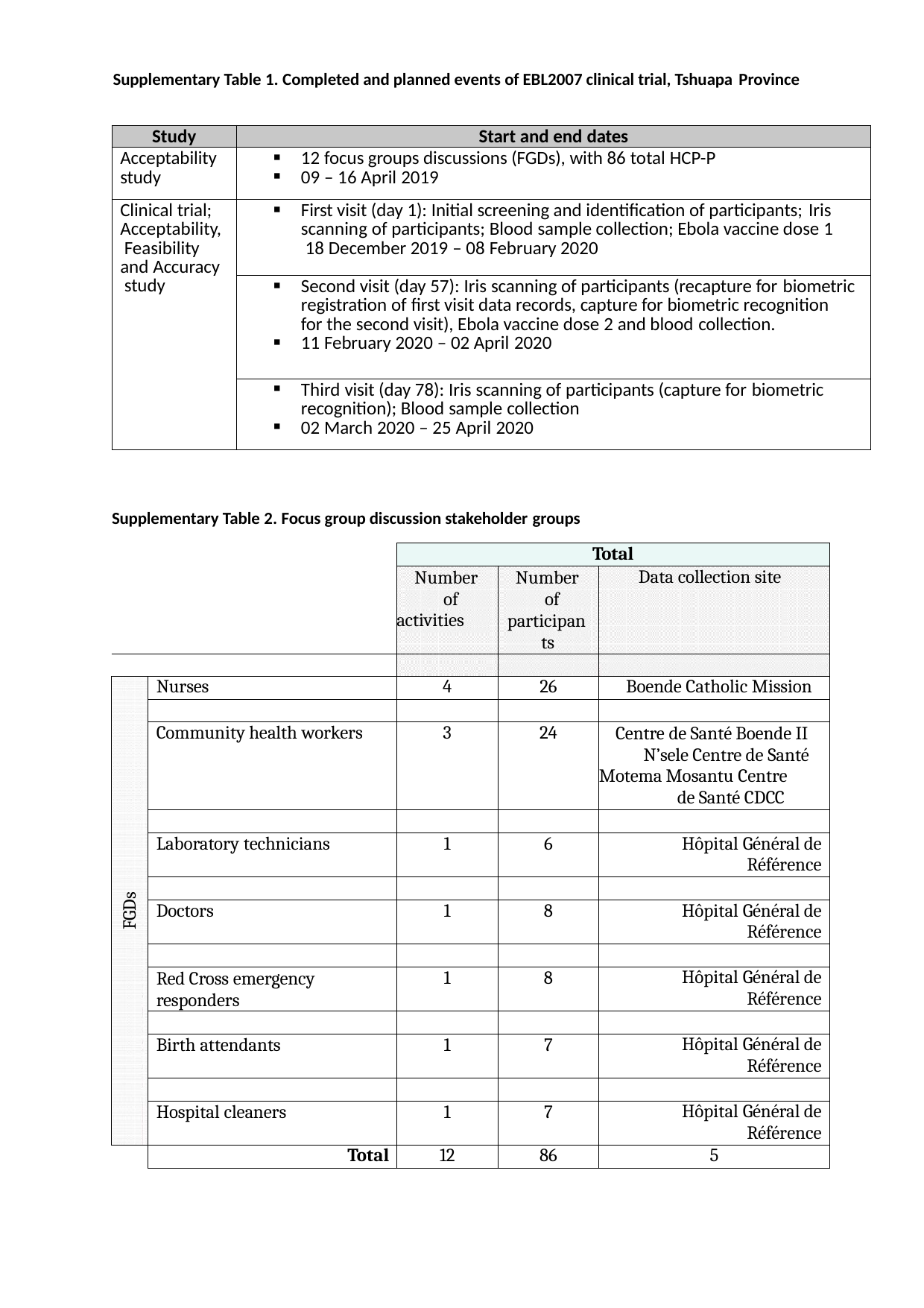

Supplementary Table 1. Completed and planned events of EBL2007 clinical trial, Tshuapa Province
| Study | Start and end dates |
| --- | --- |
| Acceptability study | 12 focus groups discussions (FGDs), with 86 total HCP-P 09 – 16 April 2019 |
| Clinical trial; Acceptability, Feasibility and Accuracy study | First visit (day 1): Initial screening and identification of participants; Iris scanning of participants; Blood sample collection; Ebola vaccine dose 1 18 December 2019 – 08 February 2020 |
| | Second visit (day 57): Iris scanning of participants (recapture for biometric registration of first visit data records, capture for biometric recognition for the second visit), Ebola vaccine dose 2 and blood collection. 11 February 2020 – 02 April 2020 |
| | Third visit (day 78): Iris scanning of participants (capture for biometric recognition); Blood sample collection 02 March 2020 – 25 April 2020 |
Supplementary Table 2. Focus group discussion stakeholder groups
| | | Total | | |
| --- | --- | --- | --- | --- |
| | | Number of activities | Number of participan ts | Data collection site |
| | | | | |
| FGDs | Nurses | 4 | 26 | Boende Catholic Mission |
| | | | | |
| | Community health workers | 3 | 24 | Centre de Santé Boende II N’sele Centre de Santé Motema Mosantu Centre de Santé CDCC |
| | | | | |
| | Laboratory technicians | 1 | 6 | Hôpital Général de Référence |
| | | | | |
| | Doctors | 1 | 8 | Hôpital Général de Référence |
| | | | | |
| | Red Cross emergency responders | 1 | 8 | Hôpital Général de Référence |
| | | | | |
| | Birth attendants | 1 | 7 | Hôpital Général de Référence |
| | | | | |
| | Hospital cleaners | 1 | 7 | Hôpital Général de Référence |
| | Total | 12 | 86 | 5 |

## Slide 2
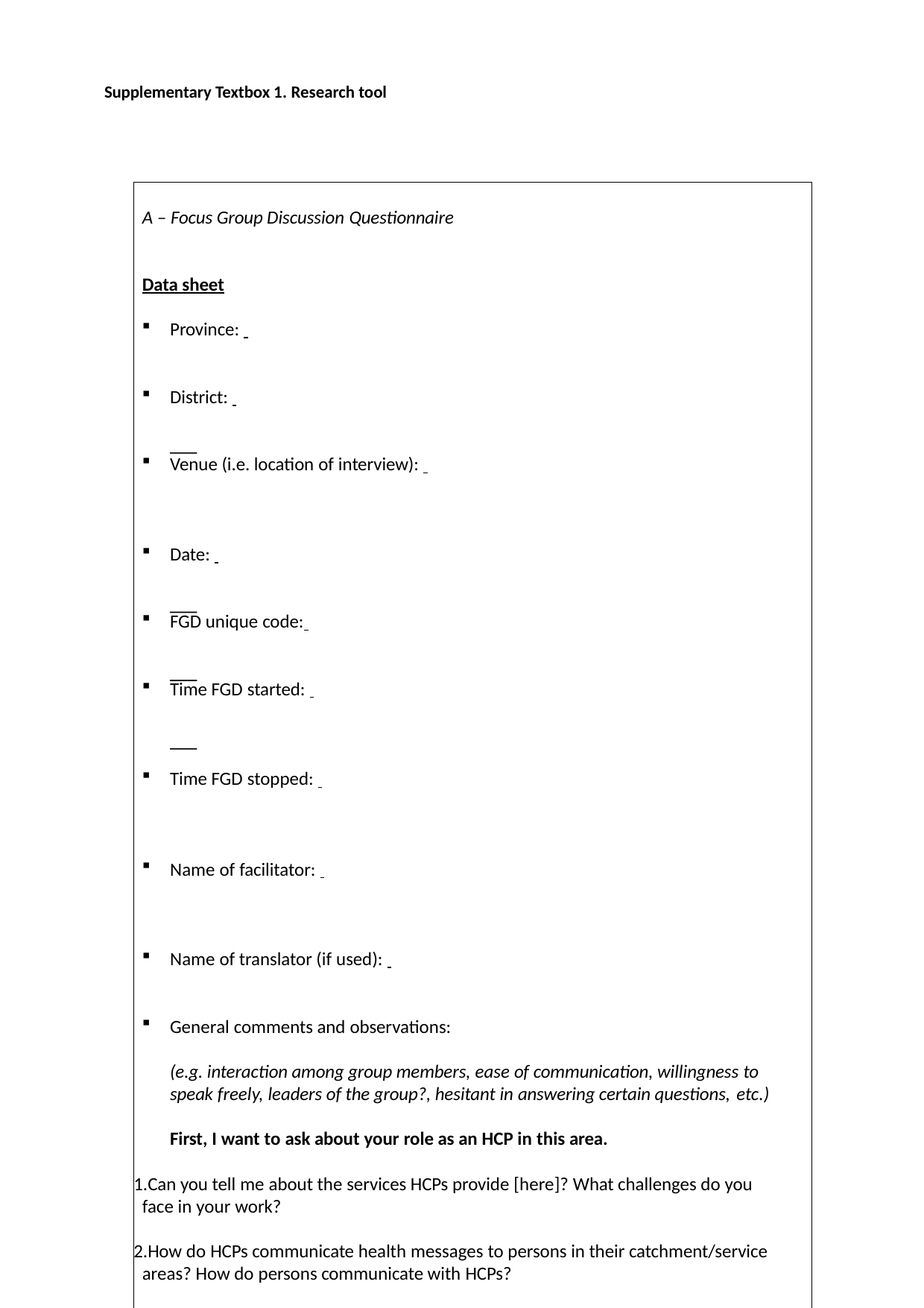

Supplementary Textbox 1. Research tool
A – Focus Group Discussion Questionnaire
Data sheet
Province:
District:
Venue (i.e. location of interview):
Date:
FGD unique code:
Time FGD started:
Time FGD stopped:
Name of facilitator:
Name of translator (if used):
General comments and observations:
(e.g. interaction among group members, ease of communication, willingness to speak freely, leaders of the group?, hesitant in answering certain questions, etc.)
First, I want to ask about your role as an HCP in this area.
Can you tell me about the services HCPs provide [here]? What challenges do you face in your work?
How do HCPs communicate health messages to persons in their catchment/service areas? How do persons communicate with HCPs?
Probes:

## Slide 3
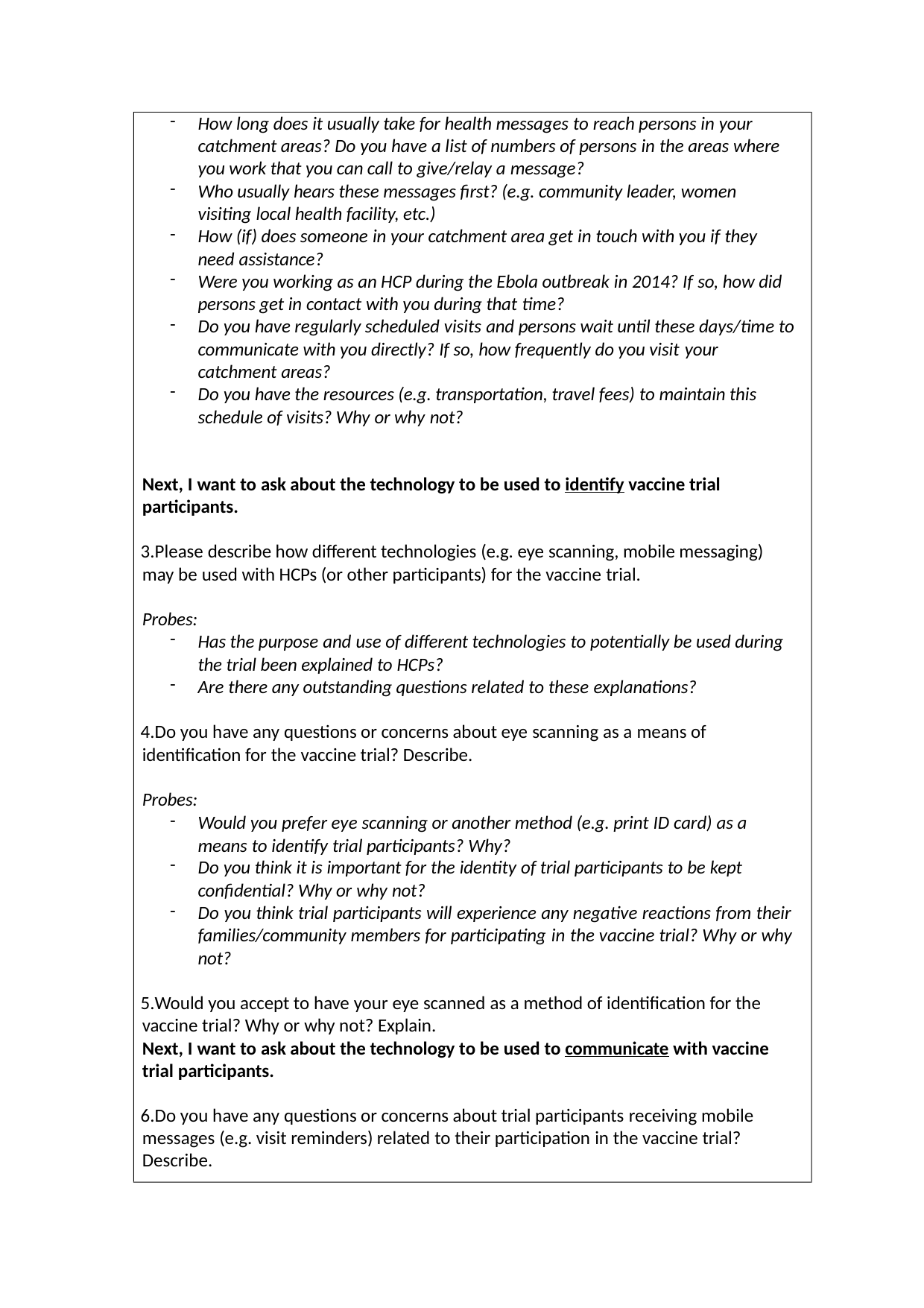

How long does it usually take for health messages to reach persons in your catchment areas? Do you have a list of numbers of persons in the areas where you work that you can call to give/relay a message?
Who usually hears these messages first? (e.g. community leader, women visiting local health facility, etc.)
How (if) does someone in your catchment area get in touch with you if they need assistance?
Were you working as an HCP during the Ebola outbreak in 2014? If so, how did persons get in contact with you during that time?
Do you have regularly scheduled visits and persons wait until these days/time to communicate with you directly? If so, how frequently do you visit your
catchment areas?
Do you have the resources (e.g. transportation, travel fees) to maintain this schedule of visits? Why or why not?
Next, I want to ask about the technology to be used to identify vaccine trial participants.
Please describe how different technologies (e.g. eye scanning, mobile messaging) may be used with HCPs (or other participants) for the vaccine trial.
Probes:
Has the purpose and use of different technologies to potentially be used during the trial been explained to HCPs?
Are there any outstanding questions related to these explanations?
Do you have any questions or concerns about eye scanning as a means of identification for the vaccine trial? Describe.
Probes:
Would you prefer eye scanning or another method (e.g. print ID card) as a means to identify trial participants? Why?
Do you think it is important for the identity of trial participants to be kept
confidential? Why or why not?
Do you think trial participants will experience any negative reactions from their families/community members for participating in the vaccine trial? Why or why not?
Would you accept to have your eye scanned as a method of identification for the vaccine trial? Why or why not? Explain.
Next, I want to ask about the technology to be used to communicate with vaccine trial participants.
Do you have any questions or concerns about trial participants receiving mobile messages (e.g. visit reminders) related to their participation in the vaccine trial? Describe.

## Slide 4
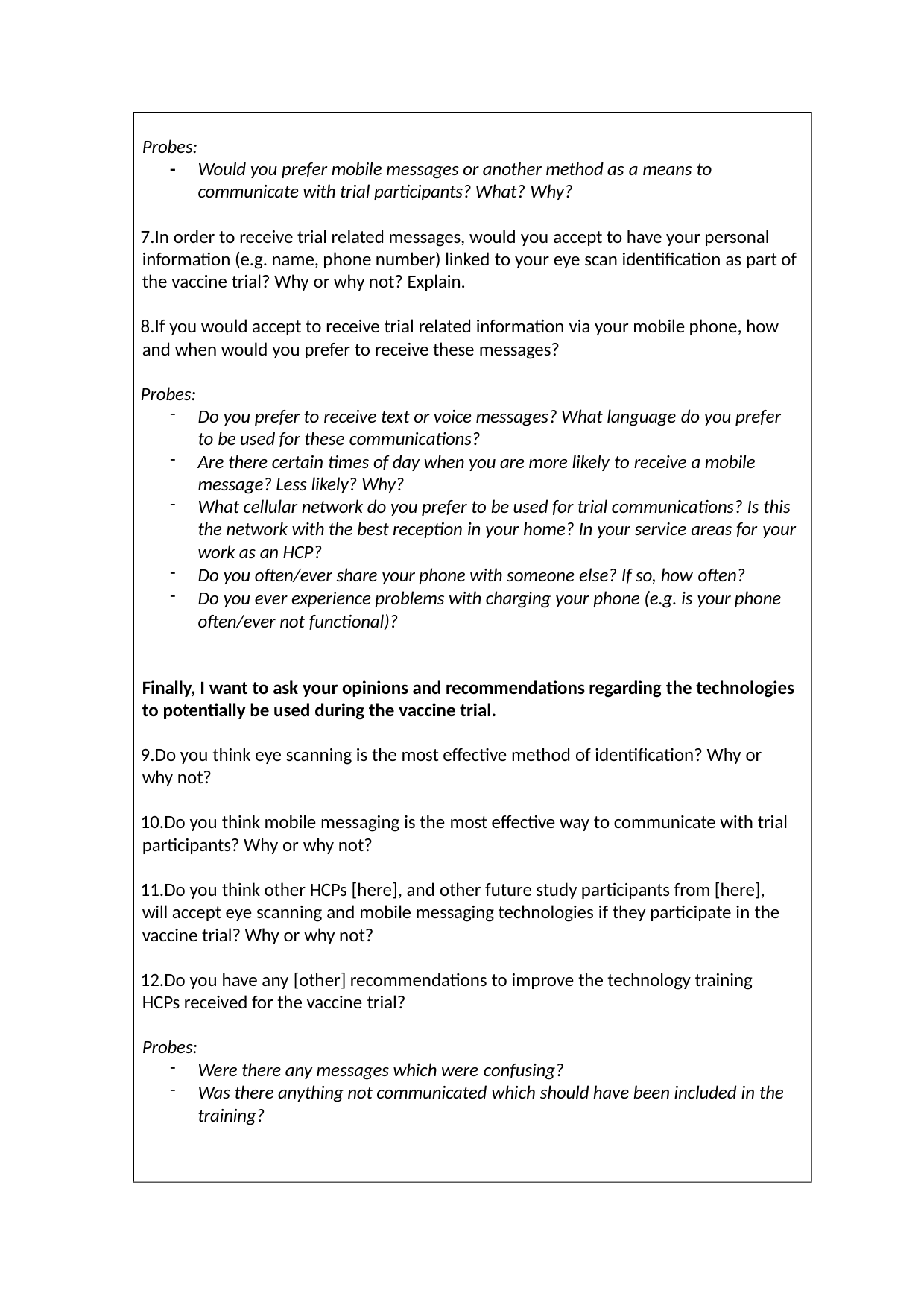

Probes:
-	Would you prefer mobile messages or another method as a means to communicate with trial participants? What? Why?
In order to receive trial related messages, would you accept to have your personal information (e.g. name, phone number) linked to your eye scan identification as part of the vaccine trial? Why or why not? Explain.
If you would accept to receive trial related information via your mobile phone, how and when would you prefer to receive these messages?
Probes:
Do you prefer to receive text or voice messages? What language do you prefer to be used for these communications?
Are there certain times of day when you are more likely to receive a mobile message? Less likely? Why?
What cellular network do you prefer to be used for trial communications? Is this the network with the best reception in your home? In your service areas for your
work as an HCP?
Do you often/ever share your phone with someone else? If so, how often?
Do you ever experience problems with charging your phone (e.g. is your phone often/ever not functional)?
Finally, I want to ask your opinions and recommendations regarding the technologies to potentially be used during the vaccine trial.
Do you think eye scanning is the most effective method of identification? Why or why not?
Do you think mobile messaging is the most effective way to communicate with trial participants? Why or why not?
Do you think other HCPs [here], and other future study participants from [here], will accept eye scanning and mobile messaging technologies if they participate in the vaccine trial? Why or why not?
Do you have any [other] recommendations to improve the technology training HCPs received for the vaccine trial?
Probes:
Were there any messages which were confusing?
Was there anything not communicated which should have been included in the training?

## Slide 5
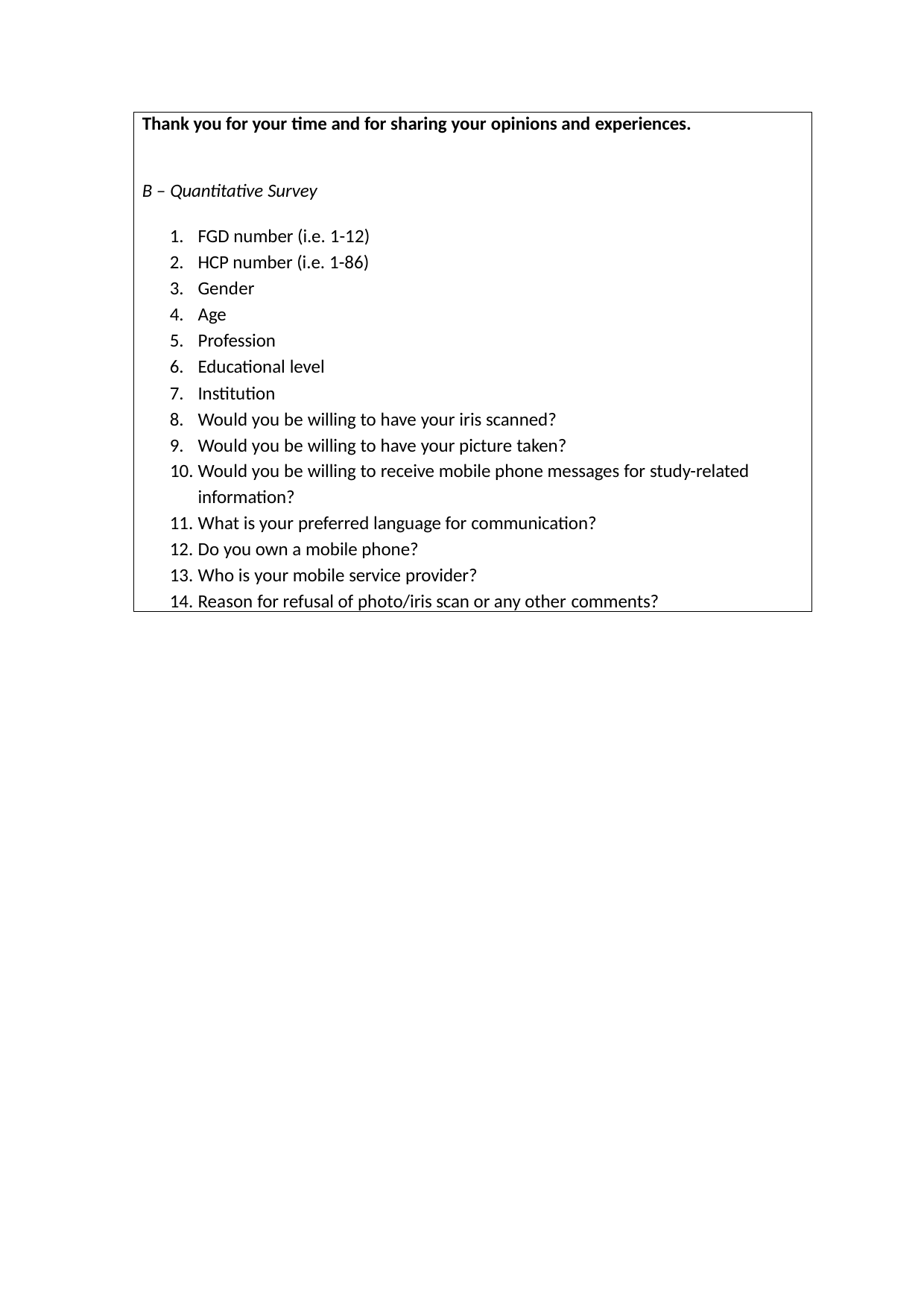

Thank you for your time and for sharing your opinions and experiences.
B – Quantitative Survey
FGD number (i.e. 1-12)
HCP number (i.e. 1-86)
Gender
Age
Profession
Educational level
Institution
Would you be willing to have your iris scanned?
Would you be willing to have your picture taken?
Would you be willing to receive mobile phone messages for study-related information?
What is your preferred language for communication?
Do you own a mobile phone?
Who is your mobile service provider?
Reason for refusal of photo/iris scan or any other comments?

## Slide 6
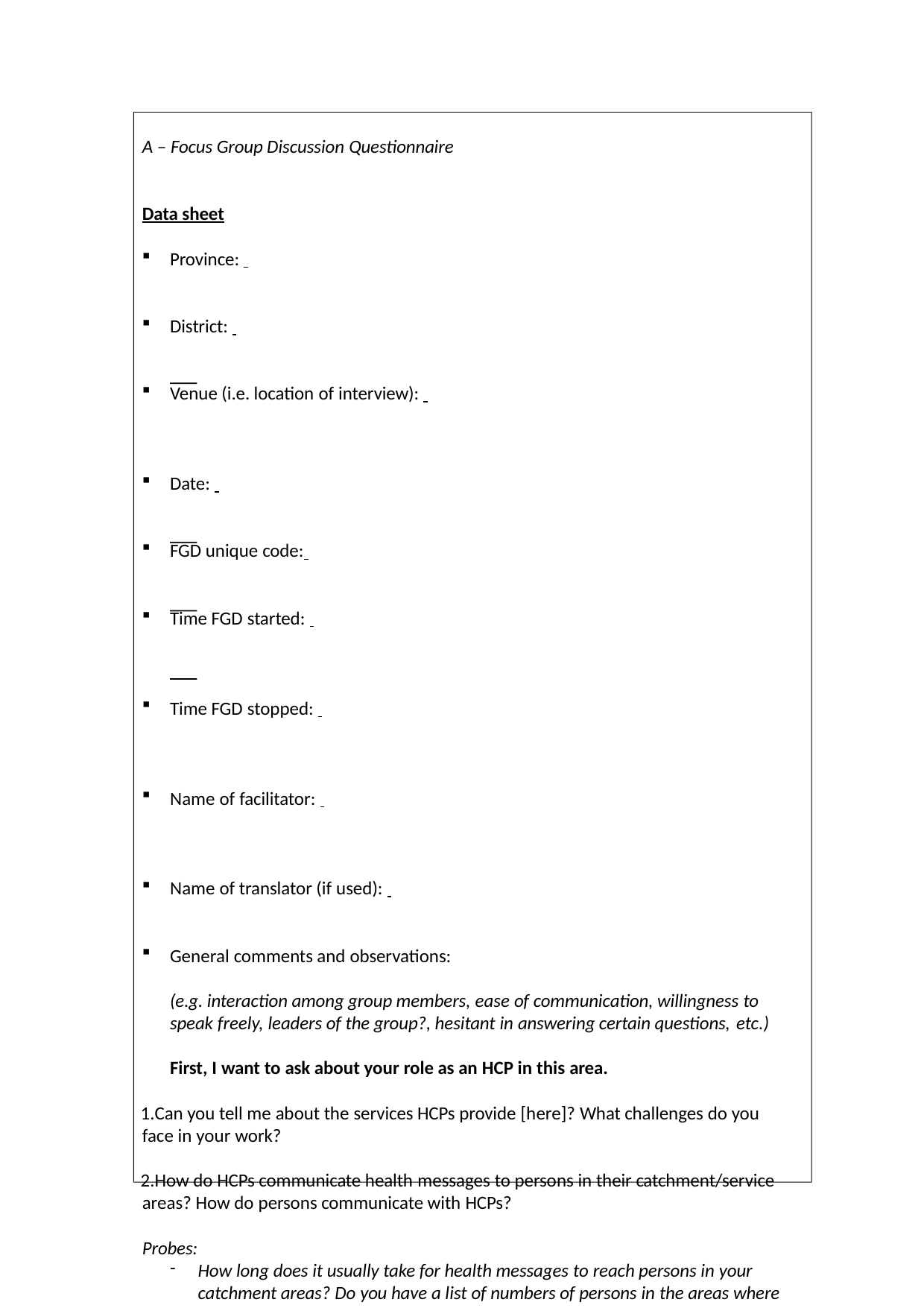

A – Focus Group Discussion Questionnaire
Data sheet
Province:
District:
Venue (i.e. location of interview):
Date:
FGD unique code:
Time FGD started:
Time FGD stopped:
Name of facilitator:
Name of translator (if used):
General comments and observations:
(e.g. interaction among group members, ease of communication, willingness to speak freely, leaders of the group?, hesitant in answering certain questions, etc.)
First, I want to ask about your role as an HCP in this area.
Can you tell me about the services HCPs provide [here]? What challenges do you face in your work?
How do HCPs communicate health messages to persons in their catchment/service areas? How do persons communicate with HCPs?
Probes:
How long does it usually take for health messages to reach persons in your catchment areas? Do you have a list of numbers of persons in the areas where you work that you can call to give/relay a message?
Who usually hears these messages first? (e.g. community leader, women visiting local health facility, etc.)

## Slide 7
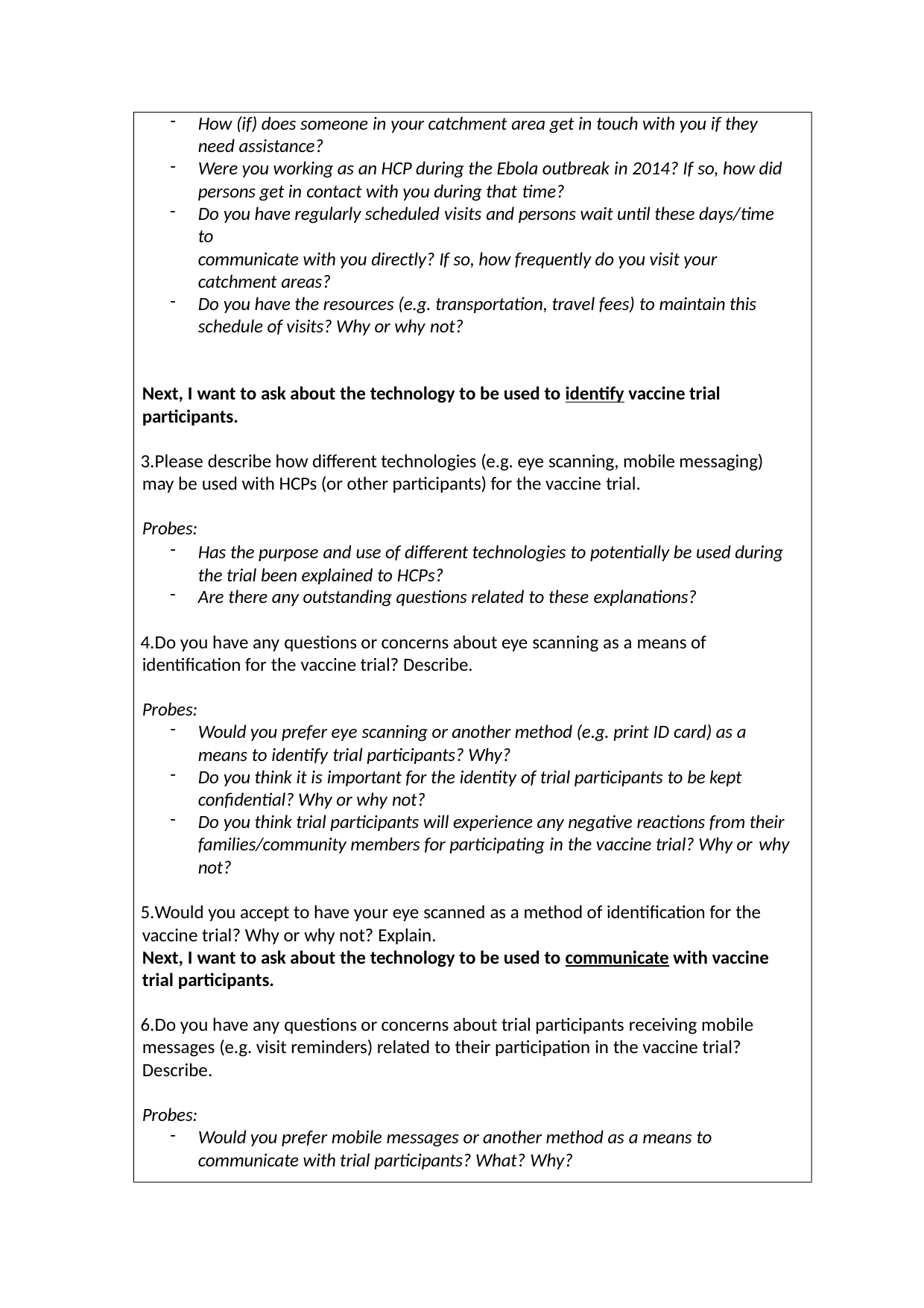

How (if) does someone in your catchment area get in touch with you if they need assistance?
Were you working as an HCP during the Ebola outbreak in 2014? If so, how did persons get in contact with you during that time?
Do you have regularly scheduled visits and persons wait until these days/time to
communicate with you directly? If so, how frequently do you visit your catchment areas?
Do you have the resources (e.g. transportation, travel fees) to maintain this schedule of visits? Why or why not?
Next, I want to ask about the technology to be used to identify vaccine trial participants.
Please describe how different technologies (e.g. eye scanning, mobile messaging) may be used with HCPs (or other participants) for the vaccine trial.
Probes:
Has the purpose and use of different technologies to potentially be used during the trial been explained to HCPs?
Are there any outstanding questions related to these explanations?
Do you have any questions or concerns about eye scanning as a means of identification for the vaccine trial? Describe.
Probes:
Would you prefer eye scanning or another method (e.g. print ID card) as a means to identify trial participants? Why?
Do you think it is important for the identity of trial participants to be kept confidential? Why or why not?
Do you think trial participants will experience any negative reactions from their families/community members for participating in the vaccine trial? Why or why
not?
Would you accept to have your eye scanned as a method of identification for the vaccine trial? Why or why not? Explain.
Next, I want to ask about the technology to be used to communicate with vaccine trial participants.
Do you have any questions or concerns about trial participants receiving mobile messages (e.g. visit reminders) related to their participation in the vaccine trial? Describe.
Probes:
Would you prefer mobile messages or another method as a means to communicate with trial participants? What? Why?

## Slide 8
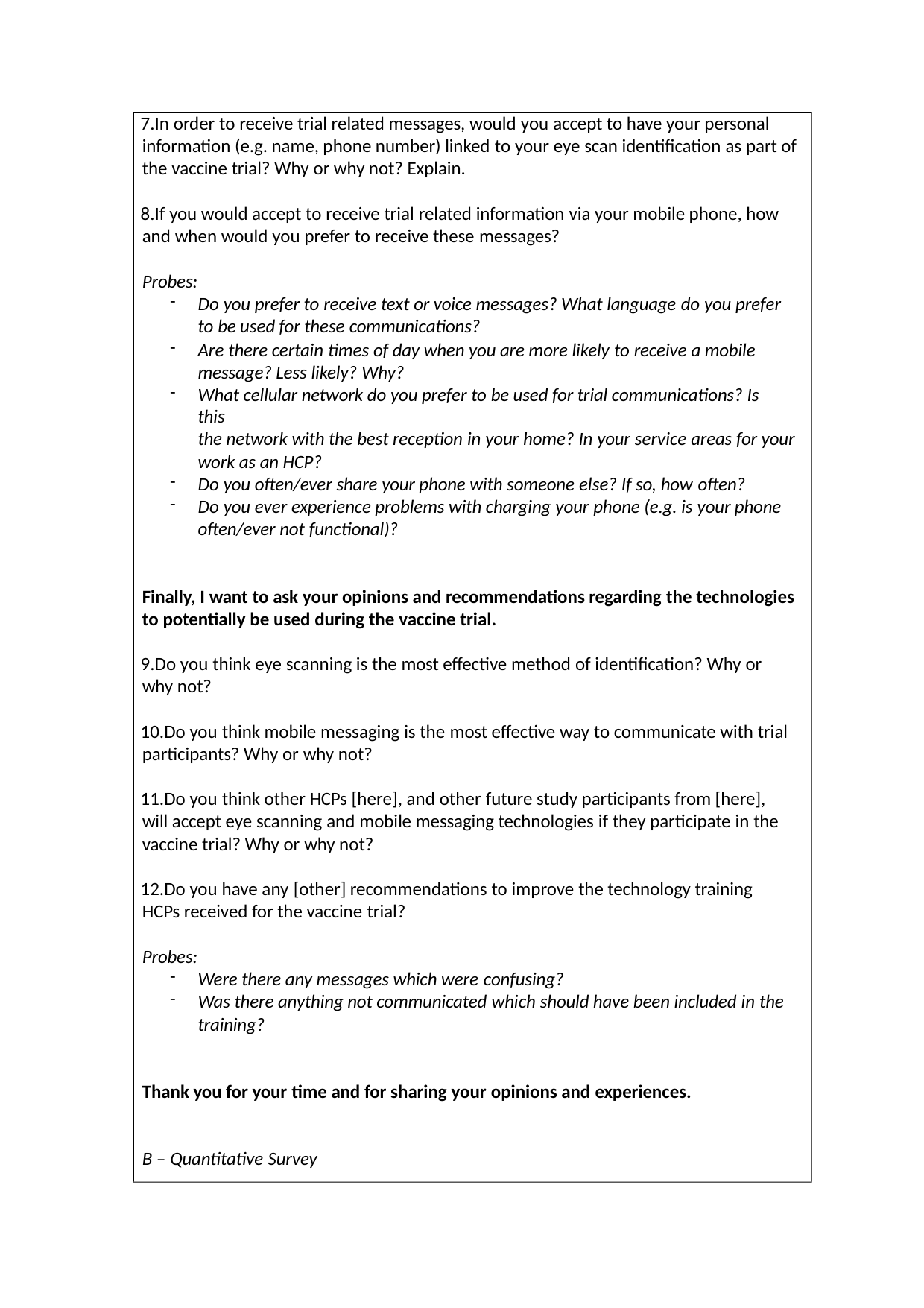

In order to receive trial related messages, would you accept to have your personal information (e.g. name, phone number) linked to your eye scan identification as part of the vaccine trial? Why or why not? Explain.
If you would accept to receive trial related information via your mobile phone, how and when would you prefer to receive these messages?
Probes:
Do you prefer to receive text or voice messages? What language do you prefer to be used for these communications?
Are there certain times of day when you are more likely to receive a mobile message? Less likely? Why?
What cellular network do you prefer to be used for trial communications? Is this
the network with the best reception in your home? In your service areas for your work as an HCP?
Do you often/ever share your phone with someone else? If so, how often?
Do you ever experience problems with charging your phone (e.g. is your phone often/ever not functional)?
Finally, I want to ask your opinions and recommendations regarding the technologies to potentially be used during the vaccine trial.
Do you think eye scanning is the most effective method of identification? Why or why not?
Do you think mobile messaging is the most effective way to communicate with trial participants? Why or why not?
Do you think other HCPs [here], and other future study participants from [here], will accept eye scanning and mobile messaging technologies if they participate in the vaccine trial? Why or why not?
Do you have any [other] recommendations to improve the technology training HCPs received for the vaccine trial?
Probes:
Were there any messages which were confusing?
Was there anything not communicated which should have been included in the training?
Thank you for your time and for sharing your opinions and experiences.
B – Quantitative Survey

## Slide 9
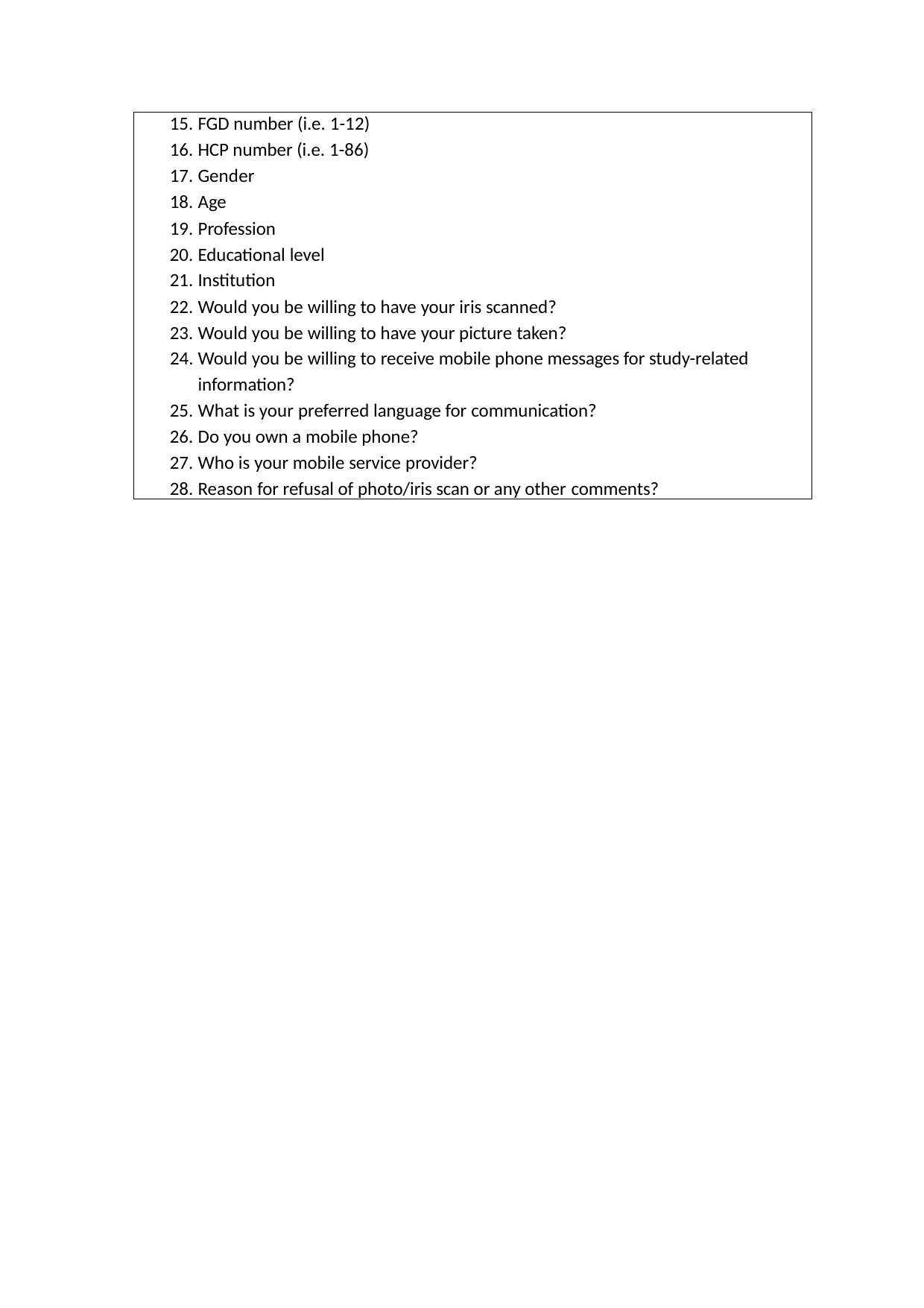

FGD number (i.e. 1-12)
HCP number (i.e. 1-86)
Gender
Age
Profession
Educational level
Institution
Would you be willing to have your iris scanned?
Would you be willing to have your picture taken?
Would you be willing to receive mobile phone messages for study-related information?
What is your preferred language for communication?
Do you own a mobile phone?
Who is your mobile service provider?
Reason for refusal of photo/iris scan or any other comments?
